# Investigation of the Photocatalytic Performance, Mechanism, and Degradation Pathways of Rhodamine B with Bi_2_O_3_ Microrods under Visible-Light Irradiation

**DOI:** 10.3390/ma17040957

**Published:** 2024-02-19

**Authors:** Dechong Ma, Jiawei Tang, Guowen He, Sai Pan

**Affiliations:** 1 College of Materials and Chemical Engineering, Hunan City University, Yiyang 413000, China; hnucityjw@163.com (J.T.); guowenh666@163.com (G.H.); saipan1985@163.com (S.P.); 2 Key Laboratory of Low Carbon and Environmental Functional Materials of College of Hunan Province, Hunan City University, Yiyang 413000, China

**Keywords:** Bi_2_O_3_ microrods, pH values, active species, degradation pathway, photodegradation mechanism

## Abstract

In the present work, the photodegradation of Rhodamine B with different pH values by using Bi_2_O_3_ microrods under visible-light irradiation was studied in terms of the dye degradation efficiency, active species, degradation mechanism, and degradation pathway. X-ray diffractometry, polarized optical microscopy, scanning electron microscopy, fluorescence spectrophotometry, diffuse reflectance spectra, Brunauer–Emmett–Teller, X-ray photoelectron spectroscopy, Fourier-transform infrared spectroscopy, UV–visible spectrophotometry, total organic carbon, and liquid chromatography–mass spectroscopy analysis techniques were used to analyze the crystal structure, morphology, surface structures, band gap values, catalytic performance, and mechanistic pathway. The photoluminescence spectra and diffuse reflectance spectrum (the band gap values of the Bi_2_O_3_ microrods are 2.79 eV) reveals that the absorption spectrum extended to the visible region, which resulted in a high separation and low recombination rate of electron–hole pairs. The photodegradation results of Bi_2_O_3_ clearly indicated that Rhodamine B dye had removal efficiencies of about 97.2%, 90.6%, and 50.2% within 120 min at the pH values of 3.0, 5.0, and 7.0, respectively. In addition, the mineralization of RhB was evaluated by measuring the effect of Bi_2_O_3_ on chemical oxygen demand and total organic carbon at the pH value of 3.0. At the same time, quenching experiments were carried out to understand the core reaction species involved in the photodegradation of Rhodamine B solution at different pH values. The results of X-ray photoelectron spectroscopy, Fourier-transform infrared spectroscopy, and X-ray diffractometer analysis of pre- and post-Bi_2_O_3_ degradation showed that BiOCl was formed on the surface of Bi_2_O_3_, and a BiOCl/Bi_2_O_3_ heterojunction was formed after acid photocatalytic degradation. Furthermore, the catalytic degradation of active substances and the possible mechanism of the photocatalytic degradation of Rhodamine B over Bi_2_O_3_ at different pH values were analyzed based on the results of X-ray diffractometry, radical capture, Fourier-transform infrared spectroscopy, total organic carbon analysis, and X-ray photoelectron spectroscopy. The degradation intermediates of Rhodamine B with the Bi_2_O_3_ photocatalyst in visible light were also identified with the assistance of liquid chromatography–mass spectroscopy.

## 1. Introduction

Rhodamine B (RhB) is a synthetic dye with a bright pink color that is often used in the textile, paint, pharmaceutical, fluorescent water tracer, food, and cosmetic industries [1,2]. It has been experimentally proven that RhB is harmful to the environment, human body, and aquatic biota due to its carcinogenic properties and non-biodegradability [3,4,5,6]. Therefore, taking into account the hazards and harmful effects of wastewater, it is worthwhile to try to remove RhB pollutants from it. The common removal methods for RhB are photocatalytic degradation [7,8,9,10,11,12,13,14,15,16,17,18,19,20,21], adsorption [22,23,24], ion exchange [25,26], electrochemical [27,28], and biological treatment [29,30]. Among these methods, photocatalytic degradation is considered to be an effective technique for the removal of RhB [31,32]. Therefore, further research is needed to develop photocatalysts for the efficient degradation of RhB.

Bi_2_O_3_ has a low band gap (2.3–3.3 eV) [33], and photogenerated electron–hole pairs (e^–^–h^+^) are formed in Bi_2_O_3_ under the action of incident light, which has been proven to be a potential photocatalyst for the removal of RhB dyes [34,35,36,37]. In the last decade, due to its low toxicity and potentially high-activity photocatalysts, Bi_2_O_3_ in a variety of forms, such as nanoparticles [38,39,40], needles [41], nanorods [42,43,44], nanobelts [45,46], thin films [47], and flower-like forms [48,49,50,51], has been synthesized through chemical precipitation [52,53], hydrothermal [54,55], microwave [56], chemical deposition [57], and electrospinning methods [58], and it has been used for the photocatalytic removal of RhB pollutants.

Recently, Bi_2_O_3_ was used as a highly efficient photocatalyst to decompose RhB in the presence of different light sources due to the different reactive oxygen species (ROSs) generated by Bi_2_O_3_ in the presence of light [59,60]. Generally, Bi_2_O_3_ can degrade RhB pollutants through the production of reactive substances (•O_−2_, •OH, h^+^, and e^−^) [61,62]. Liu et al. [63] reported that Bi_2_O_3_ nanoparticles exhibited good activity against RhB pollutants due to the microstructure of the Bi_2_O_3_ nanoparticles and the oxygen vacancy defects of the fluorite structure. Furthermore, their capture experiments confirmed that the RhB photodegradation process was contributed to by •O_−2_ and h^+^. Meena and co-authors [64] found that very small amounts of Bi_2_O_3_ nanoparticles could completely reduce RhB pollutants with an excess of NaBH_4_ within 15 min of irradiation, and the results showed that e^−^ and •O_−2_ played an important role in the photodegradation of RhB with Bi_2_O_3_ nanoparticles. Teng et al. [65] reported that both •OH and •O_−2_ radicals were important reactants in the photocatalytic process of RhB (10 mg/L) at a pH value of 10 using α-Bi_2_O_3_ nanoparticles as photocatalysts that were driven by sunlight. Bera et al. [66] found that RhB•^+^ and •OH radicals produced by RhB dye might be the main degrading agents in the degradation of RhB with α-β Bi_2_O_3_ as photocatalysts. As far as we know, however, there are few studies on the catalytic degradation of active substances of RhB with Bi_2_O_3_ at different pH values under visible light. Moreover, the possible degradation pathways of RhB with Bi_2_O_3_ microrods as photocatalysts have rarely been reported.

In this study, Bi_2_O_3_ microrods was fabricated for use as a catalytic material via chemical precipitation. The photocatalytic activity of the Bi_2_O_3_ microrods against RhB at different pH values was studied by using UV-vis spectroscopy. The structural characteristics, morphology, band gap values, PL spectra, surface chemical components, and degradation pathway of RhB with the synthesized Bi_2_O_3_ microrods were also determined by using XRD, POM, SEM, PL, DRS, BET, XPS, FT-IR, UV-Vis, and TOC analyses. In addition, the possible mechanism of photocatalytic degradation of RhB with Bi_2_O_3_ at different pH values was deduced according to the results of XRD, radical capture, FTIR, TOC, and XPS analyses. The degradation intermediates of RhB with the Bi_2_O_3_ photocatalyst in visible light were also identified with the assistance of liquid chromatography–mass spectroscopy (LC-MS), and a reasonable mechanism path was drawn according to LC-MS.

## 2. Materials and Methods

### 2.1. Materials

Though HNO_3_ was not, H_2_SO_4_, H_2_O_2_, and HCl were of technical grade and purchased from Tianjin Damao (Tianjin, China); P25 TiO_2_ nanoparticles (P25, Degussa Co., Frankfurt am Main, Germany) and other reagents, including Bi(NO_3_)_3_·5H_2_O, NaOH, and RhB were analytically pure and were also purchased from Tianjin Damao (Tianjin, China). Ascorbic acid (AC, AR), K_2_Cr_2_O_7_, isopropyl alcohol (IPA, AR), Ag_2_SO_4_, and glucose were obtained from Sinopharm (Shanghai, China) without further purification.

### 2.2. Preparation of Bi_2_O_3_ Microrods

In a typical synthesis process, 10 mL of 1 mol/L Bi(NO_3_)_3_ solution (0.02 mol HNO_3_ solution, 98 wt%) was first transferred into a three-way flask and stirred evenly. Then, NaOH (3.0 g) was dissolved in 70 mL of distilled water, slowly dripped into the above solution in a stirred state, and heated at 70 °C for 50 min. After the reaction was completed, the as-prepared yellow products were separated through vacuum filtration and washed with ethanol and deionized water; then, the samples were dried at 60 °C for 4 h.

### 2.3. Characterizations

The crystal structure and morphology of the Bi_2_O_3_ crystals were determined via X-ray diffractometry (XRD, D8 ADVANCE, Bruker AXS, Karlsruhe, Germany), polarized optical microscopy (POM, KER3101-500S, Nanjing Kell Instrument Co., Ltd., Nanjing, China), and scanning electron microscopy (SEM, Hitachi S-4800, Tokyo, Japan). The surface characteristics and chemical composition of the Bi_2_O_3_ microrods were characterized via FTIR spectrophotometry with tests ranging from 500 to 4000 cm^−1^ (FTIR, Nicolet IS5; Thermo Fisher Scientific Escalab, Waltham, MA, USA) and XPS (Hitachi AXIS SUPRA+; Hitachi High-Technologies Corp., Japan). The N_2_ adsorption–desorption curves and pore size distribution of the Bi_2_O_3_ microrods were obtained through BET (BET, 3H-2000PM1, Beishide Instrument-S&T Co., Ltd., Beijing, China). The photo-luminescent characteristics of the as-prepared Bi_2_O_3_ microrods were evaluated with a fluorescence spectrophotometer (PL, Hitachi F-7000, Japan). The degradation pathways and intermediates of RhB with Bi_2_O_3_ were evaluated by using liquid chromatography–mass spectroscopy (LC-MS) (Waters I-Class; Waterworld technology (Shanghai) Co., Ltd., Shanghai, China). The TOC of the reaction system of the RhB solution with Bi_2_O_3_ microrods was measured with a TOC analyzer (Shimadzu TOC-LCPH; Shimadzu, Tokyo, Japan). The concentration of the RhB solution and diffuse reflectance spectrum of the Bi_2_O_3_ catalyst were measured by using UV-1800 or UV-2550 (Shimadzu, Tokyo, Japan).

### 2.4. Photocatalytic Experiment on Bi_2_O_3_ Microrods

In a typical photocatalytic experiment, under laboratory conditions, 30 mg of yellow microrod-shaped Bi_2_O_3_ powders and 100 mL of 10 ppm RhB solution (pH = 3, adjusted with HCl) were added successively to a 100 mL beaker with ultrasonic dispersion for 5 min to mix the Bi_2_O_3_ powders with the RhB solution to form a uniform suspension. Then, the beaker was placed in a darkroom, and the suspension was continuously stirred for 2 h. Subsequently, the beaker was placed under a 500 W iodine–tungsten lamp for illumination with stirring for 2 h. In this process, the distance from the iodine–tungsten lamp to the surface of the RhB solution was kept at 20 cm, and 2 mL of RhB solution was taken at intervals. Then, the Bi_2_O_3_ powder in the RhB solution was removed with a centrifuge and reserved.

## 3. Results and Discussion

### 3.1. Characterization

**Figure 1:**
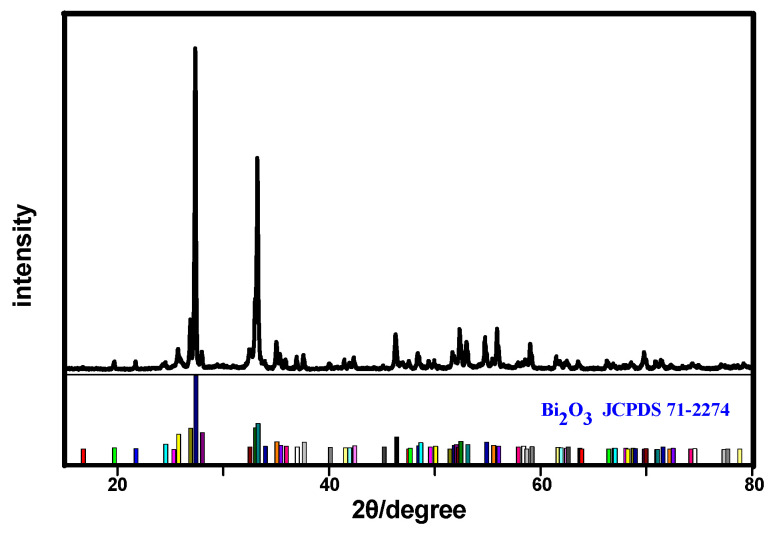
XRD pattern of the as-synthesized Bi_2_O_3_ microrods.

Figure 1 shows the XRD patterns of the Bi_2_O_3_ powders with Bi(NO_3_)_3_ as a precursor at 70 °C for 50 min in alkaline solutions. It was obvious that all of the diffraction peaks were matched with the standard card of as-synthesized Bi_2_O_3_ powders (JCPDS No. 71-2274), and no impurities were observed, which indicated that the sample obtained was high-purity Bi_2_O_3_. These results of the XRD patterns with intense and sharp diffraction peaks indicated that the as-prepared Bi_2_O_3_ sample was well crystalized.

**Figure 2:**
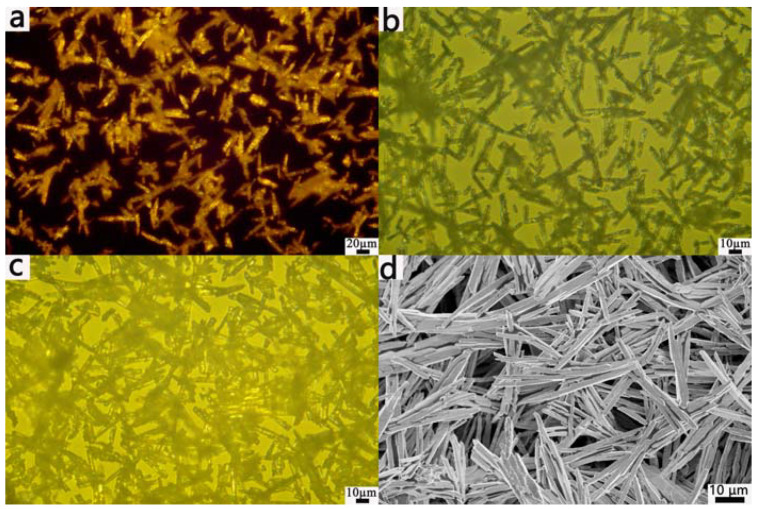
POM images of the Bi_2_O_3_ microrods with different contents of NaOH: (**a**) 0.3 g, (**b**) 0.35 g, and (**c**) 0.4 g; (**d**) SEM images of the microrods with 0.4 g of NaOH.

The concentration of the NaOH solution was a major factor affecting the growth rate, morphology, and size of the Bi_2_O_3_ crystals. While keeping other experimental parameters unchanged, we studied the effects of the morphology of Bi_2_O_3_ by changing the content of NaOH. Figure 2 shows POM and SEM images of the Bi_2_O_3_ microrods with different contents of NaOH while maintaining the volume of the NaOH solution at 70 mL and using a reaction time of 50 min. As Figure 2a shows, the Bi_2_O_3_ nanomaterials obtained were composed of Bi_2_O_3_ microrods at the NaOH content of 0.3 g. As the NaOH content was increased, the particle size of the Bi_2_O_3_ microrods increased (Figure 2b), and the scale uniformity of the Bi_2_O_3_ crystals decreased, while some of the crystals adhered to each other, as Figure 2c shows, which was consistent with the SEM images in Figure 2d. The SEM images (Figure 2d) clearly showed that the as-synthesized Bi_2_O_3_ had rod-like structures with a ratio of the length (~50 µm) to the diameter of about 10 (3–4 µm). In addition, as the reaction temperature increased, the rod-shaped Bi_2_O_3_ crystals became shorter, and the products consisted of microrods and irregular particles (see Supporting Information Figure S1).

### 3.2. Band Gap Energy Value

**Figure 3:**
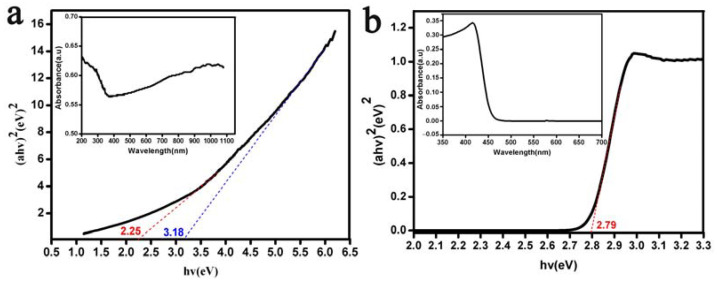
(**a**) UV-vis absorption and (**b**) UV-vis DSR spectra of the Bi_2_O_3_ microrods.

The optical band gap values of the Bi_2_O_3_ microrods were analyzed by using UV-vis DSR and UV-vis spectra based on the Kubelka–Munk method [67,68], as illustrated in Figure 3. Figure 3a shows that the variations in the band gap energy values of the Bi_2_O_3_ microrods were in the range of 2.25–3.18 eV. These results were supported by the UV–DRS spectrum analysis, as depicted in Figure 3b, where the band gap energy values of 2.79 eV obtained for the Bi_2_O_3_ microrods were consistent with the experimental findings [69,70,71]. The outcomes demonstrated that the as-prepared Bi_2_O_3_ microrods with a band gap of 2.79 eV were enough to activate photocatalysis under visible light.

### 3.3. PL Analysis of the Bi_2_O_3_ Microrods

**Figure 4:**
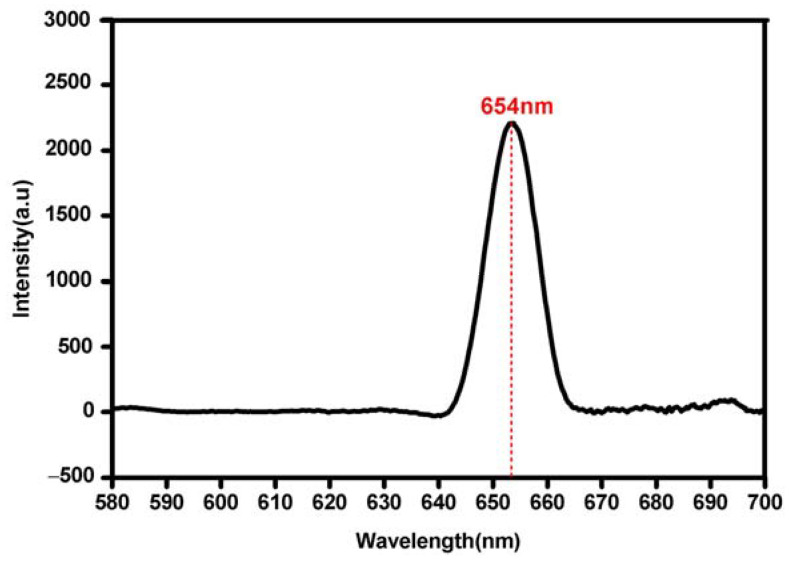
PL spectra of the as-synthesized Bi_2_O_3_ microrods.

The efficiency of photocatalysis was determined by the separation of e^–^ and h^+^. The recombination rates of both e^–^ and h^+^ in the Bi_2_O_3_ microrods were studied by using photoluminescence spectroscopy (PL) with an excitation wavelength of 434 nm, voltage of 700 V, and scan speed of 240 nm/min, as depicted in Figure 4. Figure 4 shows that a strong emission was generated at 654 nm due to the probability of charge separation and recombination between the CB and VB of the as-prepared Bi_2_O_3_ microrods. In the PL spectrum, there was a significant reduction in PL intensity below 640 nm, resulting in lower charge recombination rates and better charge carrier separation, which led to higher photocatalytic efficiency [48].

### 3.4. Adsorption and Degradation of RhB with Bi_2_O_3_ Microrods

**Figure 5:**
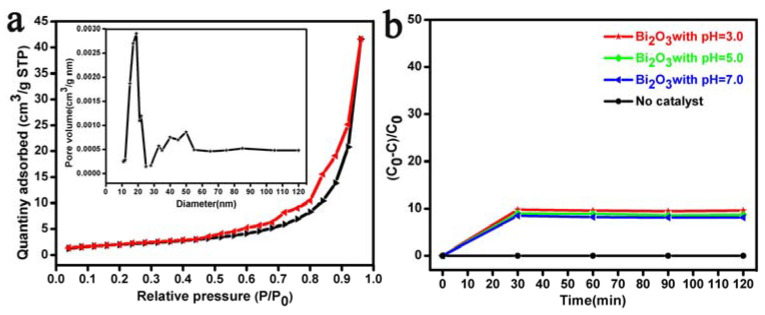
(**a**) N_2_ adsorption–desorption isotherms (the inset is their aperture distribution curve in (**a**)) of the Bi_2_O_3_ microrods; (**b**) blank experiment on the degradation of RhB solutions by Bi_2_O_3_ microrod catalysts in a dark room at a pH of 3.0–7.0.

Figure 5 shows the N_2_ adsorption–desorption isotherms, aperture distribution curve, and RhB solution photodegradation curve of the Bi_2_O_3_ microrods (see Figure 2d) at different pH values in a dark room. Figure 5a shows that the Bi_2_O_3_ microrods are a mesoporous material with a type IV and H 3 hysteresis loop [72]. BET analysis revealed that the BET surface area, pore volume, and average pore size of the Bi_2_O_3_ microrods were 4.7846 m^2^/g, 0.012 cm^3^/g, and 10.032 nm, respectively. These results confirmed that the large surface area and pore volume of the Bi_2_O_3_ microrods could expose more active sites, which was beneficial for the subsequent adsorption and photocatalysis of RhB. Figure 5b shows the blank experimental results of RhB degradation when catalyzed at different pH values in a dark room. As shown in Figure 5b, in the absence of a catalyst, the degradation of RhB could be ignored in the presence of darkness for 120 min. The curves plotted in the presence of the Bi_2_O_3_ microrod catalysts at the pH values of 3.0, 5.0, and 7.0 showed only 9.4%, 8.6%, and 8.1% decolorization after 120 min in the dark, respectively, confirming that the decolorization of RhB solutions was dominated by surface adsorption.

**Figure 6:**
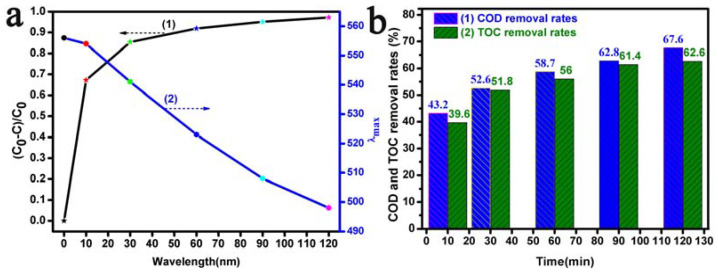
(**a**) (1) Degradation efficiencies and (**a**) (2) λ_max_ shifts; (**b**) efficiency of the removal of COD and TOC from RhB solutions with Bi_2_O_3_ microrods as photocatalysts in a typical photocatalytic experiment at a pH of 3.0.

The photocatalytic properties of RhB with the Bi_2_O_3_ microrods under visible light at a pH of 3.0 are shown in Figure 6. Figure 6a summarizes the degradation efficiencies and λ_max_ shifts (maximum wave peak displacement of RhB) of RhB with the irradiation time when using the Bi_2_O_3_ microrods in a typical photocatalytic experiment at a pH of 3.0. The maximum peak of RhB shifted blue, and the maximum absorbance gradually decreased with the increase in the illumination time, as shown in Supplementary Figure S2. After 120 min of irradiation, 97.2% degradation of RhB was achieved with the Bi_2_O_3_ microrods as photocatalysts, as shown in Figure 6a(1). Compared with hydrogen-peroxide-activated commercial P25 TiO_2_, the degradation efficiency of RhB with P25 TiO_2_ under visible light was only 55.4%, as shown in Supplementary Figure S3; therefore, the as-prepared Bi_2_O_3_ microrod catalysts were also suitable for commercial application. According to Figure 6a(2), the maximum absorption peak varied gradually from 554 to 498 nm with the prolongation of the visible-light exposure time, and the hypochromic shifts of λ_max_ were caused by the deethylation of RhB, which was confirmed by the FTIR spectra before and after RhB degradation (see Supplementary Figure S4). As shown by the decolorization of the RhB dyes, it is possible that other colorless organic molecules were formed during the degradation process, but this was not identified in the decolorization reaction. The mineralization of RhB using Bi_2_O_3_ was confirmed by the amounts of TOC and COD remaining in the decolorized RhB solutions; these were detected using a TOC analyzer and the common volumetric method, respectively. The removal efficiencies of COD and TOC in the degraded RhB solution were 67.6% and 62.6%, respectively, after 120 min treatment, as seen in Figure 6b(1,2). According to the results of the TOC analysis (Figure 6b(2)), the TOC removal efficiency increased with the extension of the illumination time, and more than 62.6% of the carbon in the RhB solution produced CO_2_ products [73,74].

**Figure 7:**
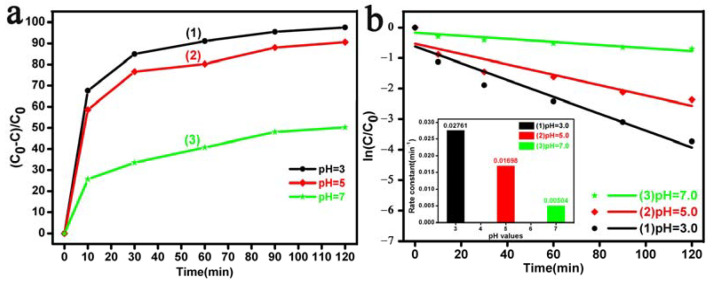
(**a**) The degradation rate and (**b**) corresponding pseudo-first-order kinetic data (the variation of the rate constant is presented in the lower inset) of RhB using the as-prepared Bi_2_O_3_ microrods as a photocatalyst at the pH values of (1) 3.0, (2) 5.0, and (3) 7.0.

The concentration of H^+^ ions in the solution is another key factor for the photodegradation of RhB dyes. Plots of the pH dependence of RhB degradation with different irradiation times are depicted in Figure 7. It can be seen in Figure 7a that the Bi_2_O_3_ microrod sample showed different photodegradation activity at different pH values. Within 120 min of irradiation, the degradation percentage of RhB in the environments of the pH value of 3.0, pH value of 5.0, and pH value of 7.0 was 97.2%, 90.6% and 50.2%, respectively. Figure 7b shows that the rate constant values (min^−1^) were 0.02761, 0.01698, and 0.00504 at the pH values of 3.0, 5.0, and 7.0 respectively. The rate constant values exhibited a maximum at the pH value of 3.0, as seen in the lower inset of Figure 7b. Greater RhB degradation at a lower pH value could be seen in this result, and this was attributed to the increased formation and accumulation of H_2_O_2_ and •OH radicals at acidic pH levels [66,75], which led to an increase in the degradation rate of RhB, resulting in a higher degradation rate higher H^+^ concentration than those at neutral levels (pH = 7.0). The results of the pH dependence experiments showed that the as-prepared Bi_2_O_3_ microrods exhibited good photocatalytic performance for RhB removal at a higher H^+^ concentration.

**Figure 8:**
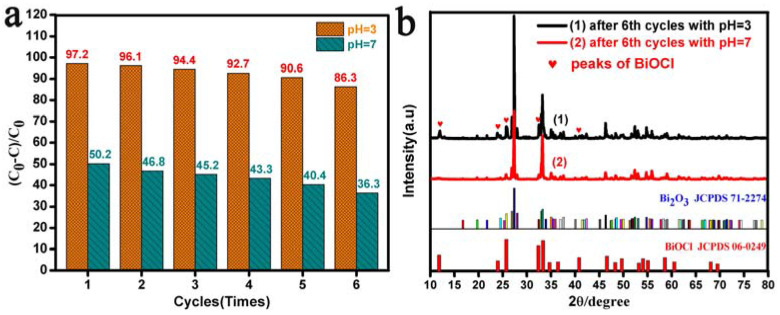
(**a**) Cycling experiments and (**b**) XRD images of the Bi_2_O_3_ microrods after the sixth cycle of degradation of RhB with pH values of 3.0 and 7.0.

Figure 8 reveals the visible-light photodegradation percentage of RhB with the Bi_2_O_3_ microrod catalyst for six cycles and the XRD patterns of the Bi_2_O_3_ microrods after six degradation cycles at different pH values. Clearly, the degradation efficiency of RhB dropped from 10.9% and 13.9% to 86.3% and 36.3% after six repetitions at the pH values of 3.0 and 7.0, respectively, as depicted in Figure 8a. Further, in order to verify the stability of the Bi_2_O_3_ microrods, XRD was employed to analyze their structure after the sixth cycle of degradation at pH values of 3.0 and 7.0, respectively. The XRD patterns of the Bi_2_O_3_ microrods after six trials at a pH value of 3.0 showed that their purity was relatively low, and several peaks of BiOCl appeared (marked with solid red hearts) in the XRD pattern in Figure 8b(1). The main reason was that the Bi_2_O_3_ microrods were dissolved by HCl during the photodegradation of RhB in the acidic environment, and BiOCl was formed on the surface of the Bi_2_O_3_ microrods. According to the descriptions in Figure 8b(2), all of the diffraction peaks of the Bi_2_O_3_ microrods after the sixth cycle of degradation at a pH value of 7.0 were matched with the standard card of the Bi_2_O_3_ microrods (JCPDS No. 71-2274), which indicated that the degraded Bi_2_O_3_ microrods retained a relatively high purity.

### 3.5. XPS and FTIR Analyses of the Bi_2_O_3_ Microrods

**Figure 9:**
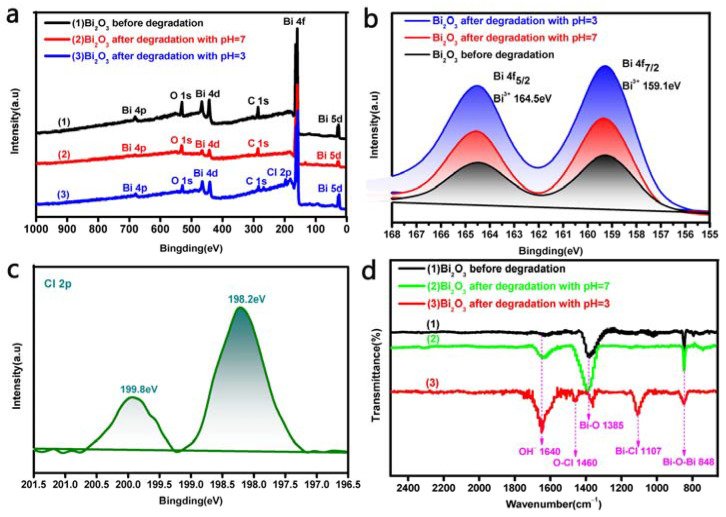
(**a**–**c**) The results of XPS and (**d**) FTIR analysis of pre- and post-photodegradation Bi_2_O_3_ microrods.

The elements and chemical components of pre- and post-photocatalytic degradation Bi_2_O_3_ microrods at different pH values were analyzed using XPS and FTIR determination, and the results are displayed in Figure 9. The elements of C, Bi, and O can be observed in full-scan spectrum shown in Figure 9a, which indicates that these three elements coexisted in Bi_2_O_3_ before and after degradation. The presence of C may have been introduced into the environment during sample preparation [76,77]. A new element, Cl, was observed in the Bi_2_O_3_ after photodegradation at a pH value of 3.0, as shown in Figure 9a(3). High-resolution XPS (HR-XPS) analysis of Bi4f in the pre- and post-photocatalytic degradation Bi_2_O_3_ samples (Figure 9b) showed that two feature peaks of the binding energies of 159.1 and 164.5 eV corresponded to Bi 4f_7/2_ and Bi 4f_5/2_ of the trivalent bismuth ion (Bi^3+^), respectively [78,79]. Figure 9c displays the HR-XPS analysis of the post-degradation Bi_2_O_3_ microrods, from which we can see that the two highest-intensity peaks located at around 199.8 and 198.2 eV corresponded to Cl 2p_1/2_ and Cl 2p_3/2_ in the region of Cl 2p, respectively, which demonstrated that BiOCl was easily produced on the surface of Bi_2_O_3_ after degradation in the acidic environment [80].

The structural characteristics of Bi_2_O_3_ microrods pre- and post-degradation at different pH values were further determined via FTIR analysis, and the results are illustrated in Figure 9d. Figure 9d(1,2) show that the spectrum of pre- and post-degradation Bi_2_O_3_ at the pH value of 7.0 displayed two typical adsorption peaks at about 1385 and 848 cm^−1^, which were related to the Bi-O bond and Bi-O-Bi bond stretching vibration and symmetrical stretching, respectively [81,82,83]. The FTIR spectra after Bi_2_O_3_ degradation at a pH value of 3.0 shown in Figure 9d(3) demonstrate that the adsorption peaks of 1460 cm^–1^ and 1107 cm^–1^ in the degraded Bi_2_O_3_ microrods were caused by O-Cl and Bi-Cl bond vibrations [84,85], respectively. In conclusion, BiOCl crystals were easily produced on the surface of the Bi_2_O_3_ crystals under acidic conditions based on the results of the XPS, FTIR, and XRD (Figure 8b) analyses of pre- and post-degradation Bi_2_O_3_.

### 3.6. Photodegradation Pathways of RhB with Bi_2_O_3_

**Figure 10:**
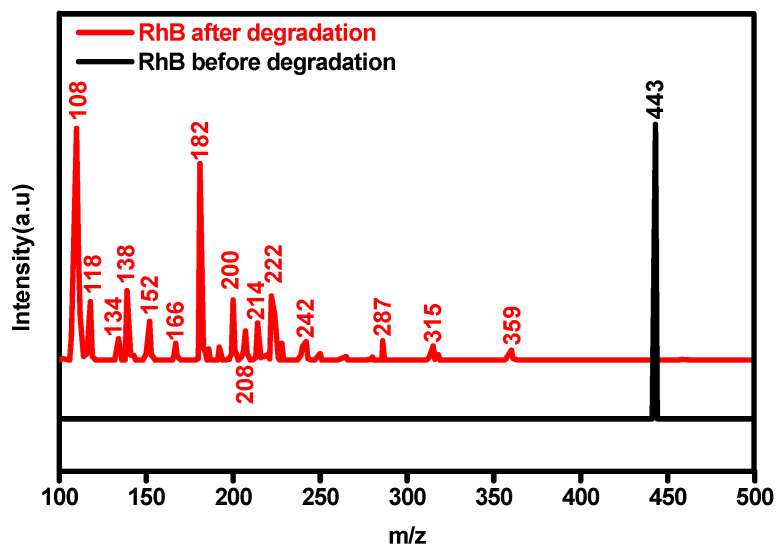
LC-MS spectra of the RhB solution before and after photodegradation with Bi_2_O_3_ microrods as a photocatalyst under irradiation.

To reveal the degradation pathway and mechanism of RhB with the Bi_2_O_3_ photocatalyst, the intermediates were determined by using LC-MS, as depicted in Figure 10. According to Figure 10, some pre- and post-degradation products were found in LC-MS, and they had strong signs at *m*/*z* 443, 359, 315, 287, 242, 222, 214, 208, 200, 182, 166, 152, 138, 134, 118, and 108.

**Figure 11:**
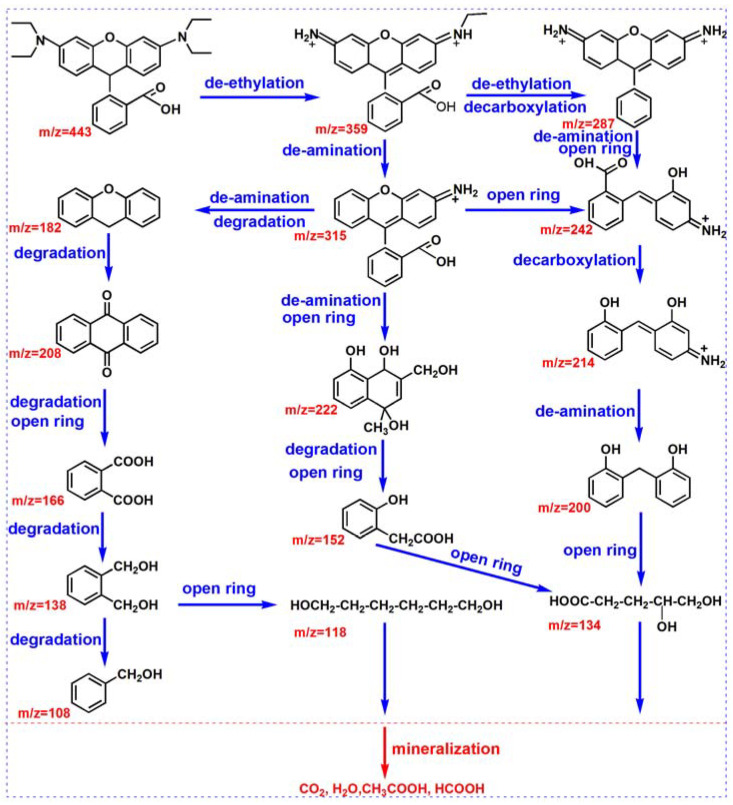
The possible degradation pathways of RhB with Bi_2_O_3_ microrods at a pH value of 3.0.

According to the intermediates analyzed with LC-MS during the reaction and in the previous literature [86,87], a possible RhB degradation pathway was proposed, as shown in Figure 11. Figure 11 displays that the degradation process of RhB mainly consisted of five steps: deethylation, decarboxylation, de-amination, ring opening, and mineralization. Initially, ethyl, carboxyl, and amino groups were removed from RhB molecules and formed multiple intermediates through ROS (•O_−2_ and •OH) attack, such as C_22_H_19_N_2_O_3_ (*m*/*z* 359), C_20_H_14_NO_3_ (*m*/*z* 315), C_19_H_15_N_2_O (*m*/*z* 287), C_14_H_12_NO_3_ (*m*/*z* 242), C_12_H_14_O_4_ (*m*/*z* 222), C_13_H_12_NO_2_ (*m*/*z* 214), C_14_H_8_O_2_ (*m*/*z* 208), C_13_H_12_O_2_ (*m*/*z* 200), and C_13_H_10_O (*m*/*z* 182). Then, there was a key ring-opening reaction, where the ring-opening reactions fragmented the aforementioned intermediates into low-molecular-weight organics, small fatty acids, and fatty alcohols, e.g., C_8_H_6_O_4_ (*m*/*z* 166), C_8_H_8_O_3_ (*m*/*z* 152), C_8_H_10_O_2_ (*m*/*z* 138), C_5_H_10_O_4_ (*m*/*z* 134), C_6_H_14_O_2_ (*m*/*z* 118), and C_7_H_8_O (*m*/*z* 108). Finally, the small molecules were further oxidized and mineralized into CO_2_ and H_2_O.

### 3.7. Degradation Mechanism

**Figure 12:**
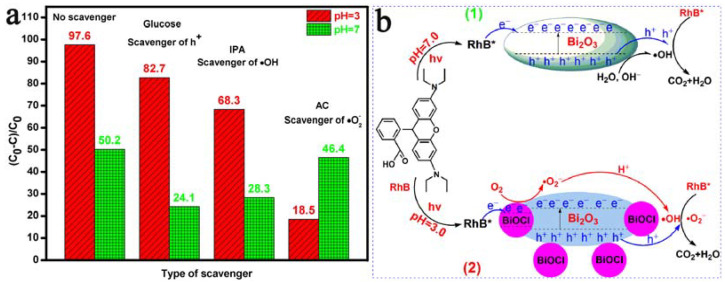
(**a**) Degradation profiles of RhB with Bi_2_O_3_ microrods without a scavenger and with glucose, IPA, and AC as scavengers of h^+^, •OH, and •O_−2_. (**b**) Degradation mechanism of Bi_2_O_3_ microrods at pH values of (1) 7.0 and (2) 3.0.

The types of active substances formed during photodegradation and the possible mechanism of the catalytic degradation of RhB with Bi_2_O_3_ microrods at the pH values of 7.0 and 3.0 were determined with a radical-trapping experiment, and the results are displayed in Figure 12a. In general, glucose, IPA, and AC were introduced as scavengers of h^+^, •OH, and •O_−2_ during photodegradation, respectively. Figure 12b(1) shows that, on the basis of the photocatalytic experiments at a pH value of 3.0, the degradation rates of RhB were 18.5% and 68.3% after the addition of AC and IPA, respectively. However, the photodegradation of RhB had little change after adding glucose, indicating that there was a small amount of h+ during the catalytic process. The experimental results indicated that •O_−2_ and •OH were the effective active substances in the photodegradation process of RhB with Bi_2_O_3_ microrods at a pH value of 3.0. Figure 12b(2) displays that hole (h^+^) or hydroxyl radicals (•OH) were the effective active substances in the photodegradation of RhB with Bi_2_O_3_ microrods at a pH value of 7.0. The photodegradation of RhB was hard to change after adding AC compared with that in the blank experiment (without a scavenger). The results indicated that the absence of •O_−2_ during the degradation of RhB and the effective active species were h^+^ and •OH in the photodegradation of RhB with Bi_2_O_3_ microrods at a pH value of 7.0.

According to the above discussion, a possible photocatalytic mechanism of RhB with Bi_2_O_3_ microrods at the pH values of 7.0 and 3.0 was proposed, as depicted in Figure 12b. As can been seen in Figure 12b(1), the mechanism of photocatalytic degradation of RhB with Bi_2_O_3_ was summarized at a pH value of 7.0. In the presence of visible light, e^−^ in the VB of the semiconducting Bi_2_O_3_ microrod photocatalyst was excited to CB, and VB produced photogenerated h^+^, which partially complexed with e^−^ of CB; then, some photogenerated h^+^ reacted with H_2_O or OH^−^ to form •OH. Thus, RhB molecules reacted with the effective active substances of h^+^ and •OH and formed multiple small intermediates, which were mineralized into CO_2_ and H_2_O. At the same time, a rational photocatalytic degradation mechanism of RhB with Bi_2_O_3_ microrods under acidic conditions was also proposed (pH value = 3.0), and a schematic is shown in Figure 12(2). During the initial degradation, the surface layer of the Bi_2_O_3_ microrods was dissolved by HCl, and a heterojunction of BiOCl/Bi_2_O_3_ was formed with a small amount of BiOCl on the surface of the Bi_2_O_3_ samples, which would facilitate the migration of photoinduced charge carriers [88]. Furthermore, the addition of HCl made it easier for O_2_ to capture the e^−^ of photocatalyst CB and produce •O_−2_, which could further convert •OH in the presence of H^+^. After multiple photodegradation cycles, the contact probability of Bi_2_O_3_ with RhB decreased with the increase in BiOCl content on the surface of Bi_2_O_3_ in the BiOCl/Bi_2_O_3_ heterostructure (Figure 8b, XRD pattern); the photocharge carrier migration was weakened, and the removal rate of RhB significantly decreased.

## 4. Conclusions

In summary, the results of the POM and SEM analyses showed that the Bi_2_O_3_ catalyst with a microrod-like structure was prepared with a chemical precipitation method. The results of the PL spectra and DRS (the band gap value of the Bi_2_O_3_ microrods is 2.79 eV) revealed that the absorption spectrum extended to the visible region, which resulted in a high separation and low recombination rate of e^−^ and h^+^. The photodegradation results of Bi_2_O_3_ clearly indicated that about 97.2%, 90.6%, and 50.2% degradation of RhB dyes was observed within 120 min at the pH values of 3.0, 5.0, and 7.0, respectively. The TOC removal efficiency increased with the extension of the illumination time, and more than 62.6% of the carbon in the RhB solution produced CO_2_ products. The experimental results indicated that •O_−2_, •OH, and h^+^ or •OH were the effective active substances in the degradation process of RhB with Bi_2_O_3_ microrods at the pH values of 3.0 and 7.0, respectively. The results also revealed that a heterojunction of BiOCl/Bi_2_O_3_ was formed with a small amount of BiOCl on the surface of Bi_2_O_3_ samples based on the results of XRD, XPS, and FTIR analysis techniques. Furthermore, the effective active substances and possible mechanisms of photocatalytic degradation of Bi_2_O_3_ at different pH values were analyzed based on the results of XRD, radical capture, FTIR, TOC, and XPS analyses. The degradation process of RhB mainly consisted of five steps: deethylation, decarboxylation, de-amination, ring opening, and mineralization.

## Data Availability

Data are contained within the article.

## Supplementary Materials

The following supporting information can be downloaded at: https://www.mdpi.com/article/10.3390/ma17040957/s1, Figure S1: POM (a–c) images of microrods obtained at different reaction temperatures: (a) 70 °C, (b) 75 °C, and (c) 80 °C; (d) SEM images of microrods at a reaction temperature of 80 °C Figure S2: UV–visible spectral changes in the RhB solution with Bi_2_O_3_ microrods as photocatalysts; Figure S3: Photodecomposition curves of RhB solutions under visible-light irradiation with P25 TiO_2_ photocatalysts; Figure S4: The FTIR spectra of pre-degradation and post-degradation RhB solution with Bi_2_O_3_ microrods as photocatalysts.
