# Supplementary material for: Investigation of the Photocatalytic Performance, Mechanism, and Degradation Pathways of Rhodamine B with Bi2O3 Microrods under Visible-Light Irradiation
Source: Materials (Basel). 2024 Feb 19;17(4):957. doi: 10.3390/ma17040957 (PMC10890279; doi:10.3390/ma17040957)
Supplement: Supplementary file 1 [file materials-17-00957-s001.zip › materials-2856143-supplementary.pdf]

Supporting Information

Investigation of the Photocatalytic Performance, Mechanism, and Degradation Pathways of Rhodamine B with Bi_2O_3 Microrods under Visible-Light Irradiation

Dechong Ma [1,2,*], Jiawei Tang [1], Guowen He [1,2] and Sai Pan [1,2]

[1] College of Materials and Chemical Engineering, Hunan City University, Yiyang 413000, China; hnucityjw@163.com (J.T.); guowenh666@163.com (G.H.); saipan1985@163.com (S.P.)

[2] Key Laboratory of Low Carbon and Environmental Functional Materials of College of Hunan Province, Hunan City University, Yiyang 413000, China

* Correspondence: madechong@hncu.edu.cn or mars.dc@163.com

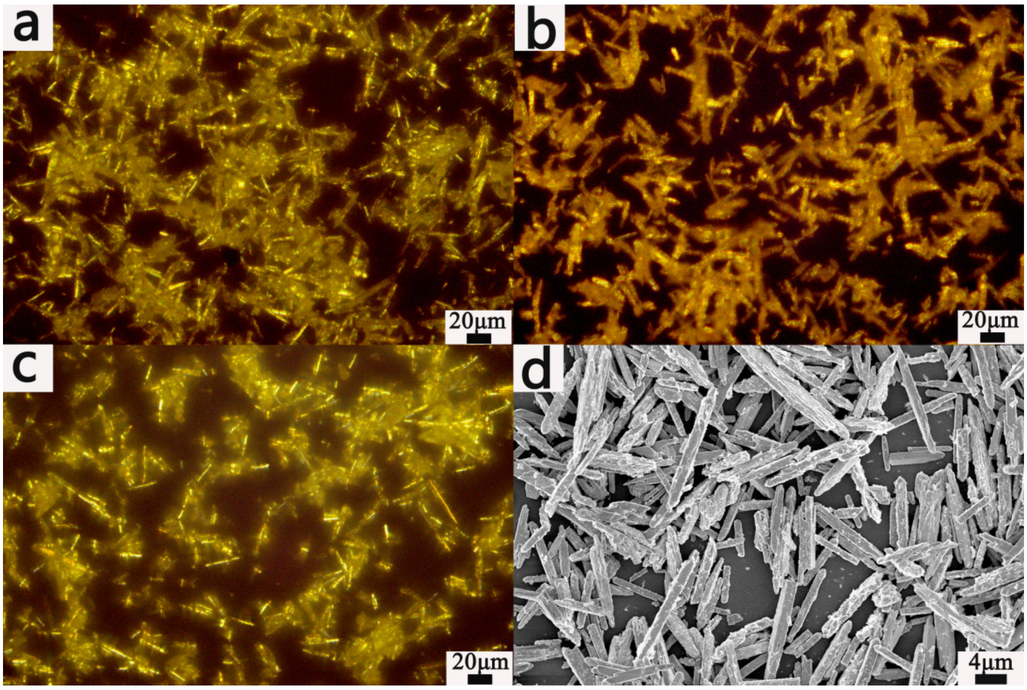

Figure S1. POM (a–c) images of microrods obtained at different reaction temperatures: (a) 70 ℃, (b) 75 ℃, and (c) 80 ℃; (d) SEM images of microrods at a reaction temperature of 80 ℃.

Figure S1 shows POM and SEM images of the Bi_2O_3 microrods prepared at different temperatures with a reaction time of 50 min. As the reaction temperature increased, the rod-shaped Bi_2O_3 crystals became shorter and had more obvious small particles on their surface, especially at the two ends of the rod, as Figure S1b and c show. Figure S1d displays an SEM image of the products prepared at 80 ℃, and we can see that the products consisted of microrods and irregular particles. The as-obtained Bi_2O_3 microrods were about 2–4 μm in diameter and 40 μm in length.

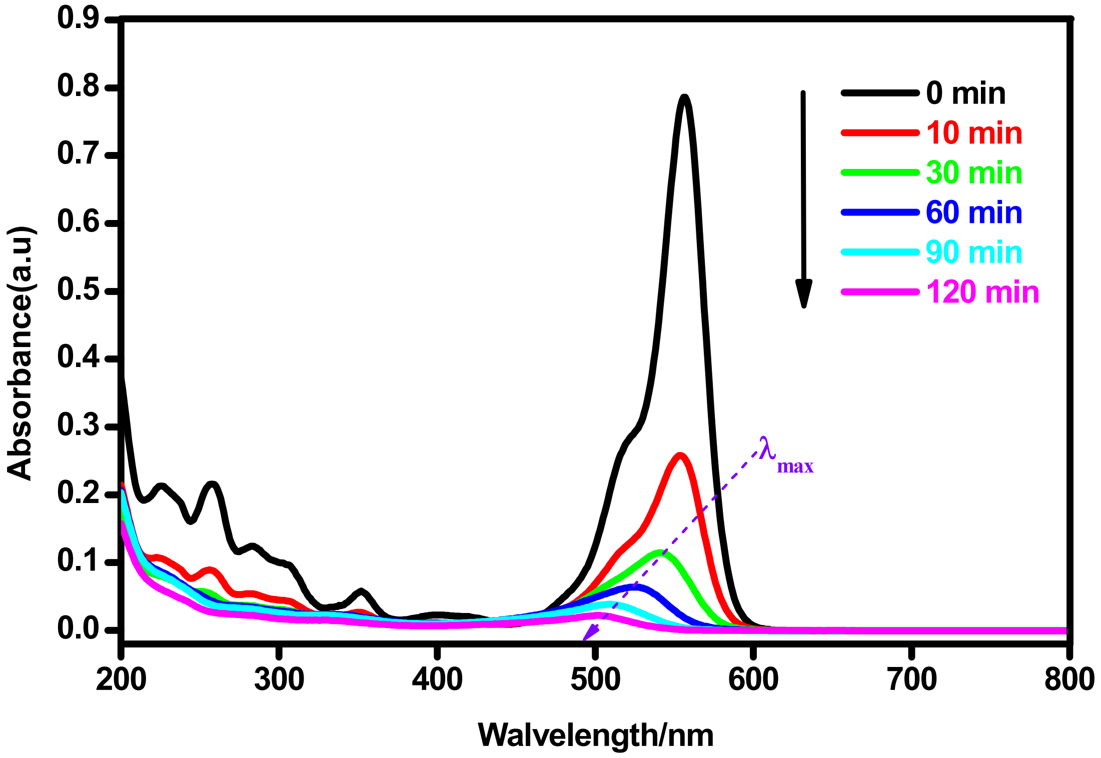

Figure S2. UV–visible spectral changes in the RhB solution with Bi_2O_3 microrods as photocatalysts.

Figure S2 shows that the maximum wave peak of RhB shifted blue, and the maximum absorbance gradually decreases with the increase in illumination time.

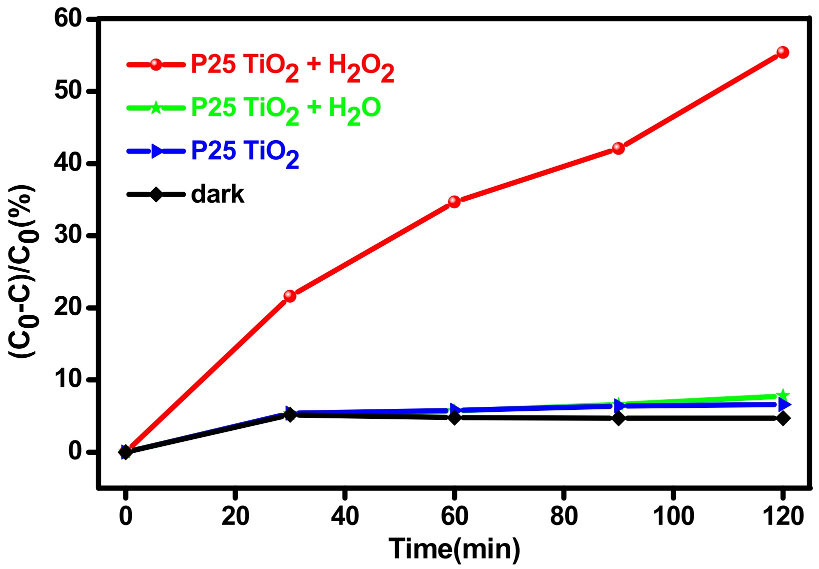

Figure S3. Photodecomposition curves of RhB solutions under visible-light irradiation with P25 TiO_2 photocatalysts.

No obvious photodegradation was detected in the contrast experiments using P25 TiO_2+H_2O and pure P25 TiO_2 under visible light, as shown in Figure S3. Remarkably, the photocatalytic degradation of RhB with the P25 TiO_2+H_2O_2 sample was efficient, and nearly 56% of the RhB was removed after 120 min of irradiation, which indicated the excellent visible-light-driven photocatalytic performance of P25 TiO_2+H_2O_2.

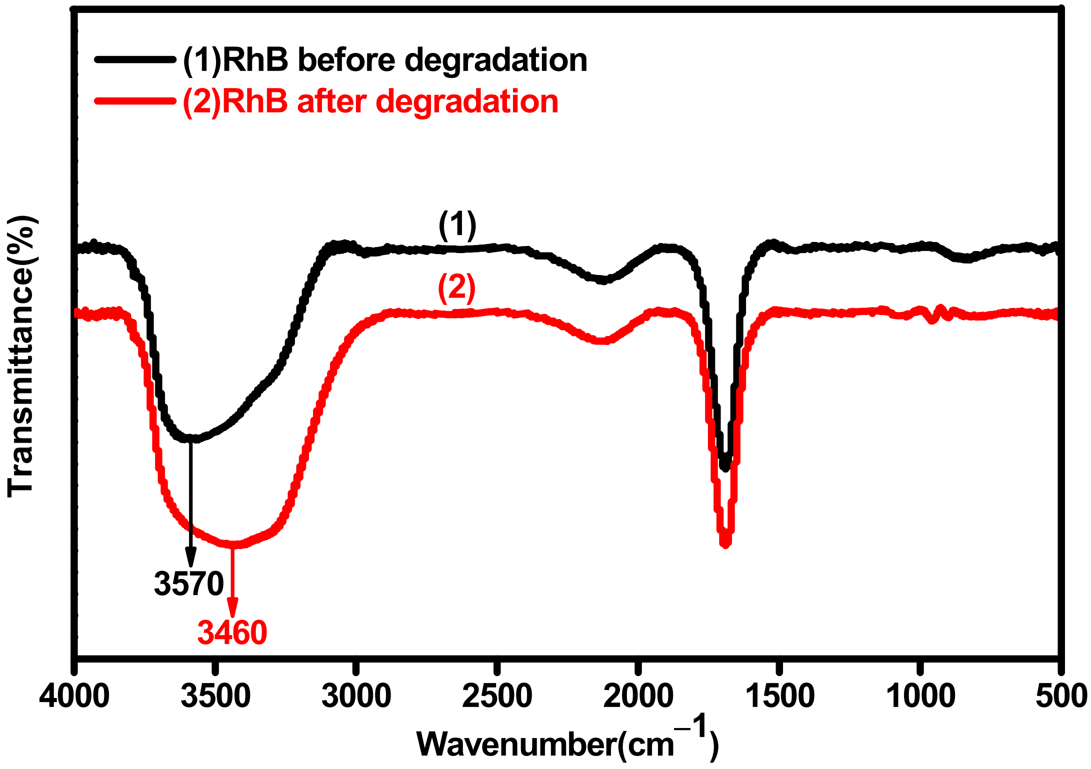

Figure S4. The FTIR spectra of pre-degradation and post-degradation RhB solution with Bi_2O_3 microrods as photocatalysts.

Comparing the FTIR spectra before and after RhB degradation, we found significant changes in the spectral range of 3600–3200 cm^{-1}, as shown in Figure S4. As shown in Figure S4, the peak of pre-degradion and post-degradation RhB showed that the absorption bands at approximately 3570 cm^{-1} and around 3460 cm^{-1} were assigned to the presence of hydrogen bonds with C–H/O–H groups in the pure state and stretching vibrations corresponding to –HN– and –HNC– groups respectively, which indicated the process of N-deethylation.
